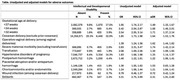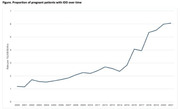# Oral Concurrent Session 5 – Hypertension: Friday, January 31, 2025 1:30 PM – 4:00 PM

**DOI:** 10.1002/pmf2.12014

**Published:** 2025-01-05

## 57 | Myo‐inositol Supplementation to Prevent Pregnancy Complications in Polycystic Ovary Syndrome: a Randomized Controlled Trial

Anne W.T. Van Der Wel^1^; Rebekka Bout‐Rebel^2^; Chryselle M.C. Frank^3^; Ruben G. Duijnhoven^1^; Bo E. Van Bree^4^; Olivier Valkenburg^4^; Salwan Al‐Nasiry^4^; Robbert H.F. van Oppenraaij^5^; Tatjana E. Vogelvang^6^; Michelle E.M.H Westerhuis^7^; Jan Peter de Bruin^8^; Hedwig P. van de Nieuwenhof^8^; Susanne C.J.P. Gielen^9^; Myrthe L. Bandell^10^; Mireille N. Bekker^11^; Maurice G.A.J. Wouters^1^; Velja Mijatovic^1^; Arie Franx^2^; Cornelis B. Lambalk^1^; Frank J.M. Broekmans^11^; Rebecca C. Painter^1^; Bart C.J.M. Fauser^12^; Joop S.E. Laven^2^; Bas B. van Rijn^13^; MYPP Investigator Group^2^

*^1^Amsterdam UMC, location University of Amsterdam, Amsterdam, Noord‐Holland; ^2^Erasmus University Medical Center, Zuid‐Holland; ^3^St. Antonius Ziekenhuis, Utrecht; ^4^Maastricht UMC+, Limburg; ^5^Maasstad Ziekenhuis, Zuid‐Holland; ^6^Het Diakonessenhuis, Utrecht; ^7^Catharina Ziekenhuis, Noord‐Brabant; ^8^Jeroen Bosch Ziekenhuis, Noord‐Brabant; ^9^Franciscus, Zuid‐Holland; ^10^Albert Schweitzer ziekenhuis, Zuid‐Holland; ^11^University Medical Center Utrecht, Utrecht; ^12^University of Utrecht and University Medical Center Utrecht, Utrecht; ^13^Eindhoven University of Technology, Noord‐Brabant*

1:30 PM ‐ 1:45 PM

**Objective**: To assess whether daily myo‐inositol supplementation reduces a composite of gestational diabetes mellitus, preeclampsia and/or preterm birth in pregnant persons with polycystic ovary syndrome.

**Study Design**: In this double‐blind, multicenter randomized placebo controlled trial, pregnant persons with polycystic ovary syndrome received daily supplementation of 4 grams of myo‐inositol, or matching placebo, from between 8 and 16 weeks gestation until delivery. The primary outcome was a composite of gestational diabetes mellitus, preeclampsia or preterm birth. Key secondary outcomes included obstetric, maternal and neonatal outcomes. The analysis was performed according to the intention‐to‐treat principle.

**Results**: A total of 464 individuals, recruited at 13 hospitals in the Netherlands, underwent randomization. Characteristics at baseline were similar between groups, except for a higher proportion of biochemical hyperandrogenism in the myo‐inositol group (29.0% vs. 18.5% in the placebo group). Two‐thirds (67.5%) of participants conceived after assisted reproductive technology. The primary outcome occurred in 56 out of 224 participants (25.0%) in the myo‐inositol group and in 61 out of 228 participants (26.8%) in the placebo group (relative risk, 0.93; 95% confidence interval, 0.68 to 1.28; P = 0.67). No substantial between‐group differences were observed for secondary outcomes, except for a higher incidence of primary caesarean section in the placebo group (11.6%, versus 5.9% in the myo‐inositol group; relative risk 0.51; 95% confidence interval 0.27 to 0.97; P< 0.05). In the preplanned sub group analysis, no interactions were observed for participants with biochemical hyperandrogenism or obesity.

**Conclusion**: Myo‐inositol supplementation in pregnant individuals with polycystic ovary syndrome does not appear to lower the incidence of a composite outcome of gestational diabetes mellitus, preeclampsia or preterm birth compared with placebo. (Funded by The Netherlands Organisation for Health Research and Development [project number 848016013]; Research with human participants [CCMO] ID NL67329.078.18)

## 58 | Nifedipine versus Labetalol for Treatment of Postpartum Hypertension: A Randomized Controlled Trial

Todd R. Lovgren^1^; Ruofan Yao^2^; Brendan D. Connealy^3^; Robert Bonebrake^1^; Hemant Satpathy^1^; Emily Patel^1^; Matthew Brady^1^; Joshua Dahlke^4^

*^1^Nebraska Methodist Health System, Elkhorn, NE; ^2^Loma Linda University Health, Loma Linda, CA; ^3^Methodist Women's Hospital, Omaha, NE; ^4^Nebraska Methodist Hospital, Elkhorn, NE*

1:45 PM ‐ 2:00 PM

**Objective**: Postpartum hypertensive disease is a significant cause of maternal morbidity and mortality, and the optimal treatment is uncertain. The purpose of this study was to determine if medication choice affects the incidence of postpartum readmission for hypertensive complications.

**Study Design**: This is a single institution, open‐label randomized trial comparing nifedipine and labetalol for treatment of postpartum hypertension. Patients with at least two hypertensive blood pressures (>140 systolic or >90 diastolic) four hours apart during their admission for delivery were identified. Once enrolled, patients were randomized to nifedipine or labetalol, irrespective of any prior treatment. The primary outcome was readmission for postpartum treatment of hypertension. Clinical characteristics evaluated included, frequency of severe hypertension, acute treatment of hypertension during hospitalization, magnesium sulfate use, and hypertension within 12 hours of discharge. Trial Registration: NCT05309460

**Results**: During the study period, 3015 patients met inclusion criteria and 324 patients were enrolled and randomly allocated to nifedipine or labetalol. The labetalol group had a higher BMI (35.3 vs 33.5), later GA at delivery (38 vs 37 weeks) and higher prevalence of CHTN (n = 17 vs 7). After controlling for the significant clinical characteristics, the incidence of readmission was 88% lower in the nifedipine arm (1.2% vs 8.1%); aOR of 0.12 (95% confidence interval [CI] 0.026 to 0.57). The frequency of severe HTN, acute treatment of HTN during hospitalization, use of magnesium sulfate and HTN within 12 hours of discharge was similar between groups.

**Conclusion**: The primary outcome was statistically significant, representing a significant decrease in readmission in those taking nifedipine. Coupled with published observational studies demonstrating similar results, this study supports Nifedipine as the initial agent of choice in the ongoing management of hypertension in the postpartum period.

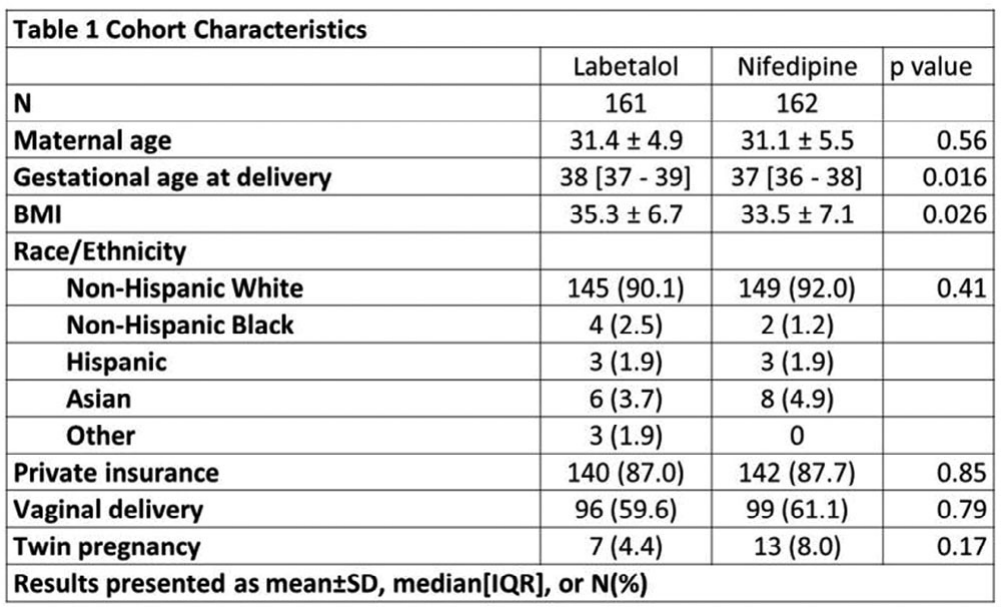

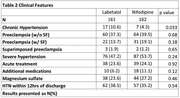

## 59 | Defining Postpartum Cardiovascular Physiology: Blood Pressure Trajectories in Patients at risk for New‐Onset Postpartum Hypertension

Ukachi N. Emeruwa^1^; Minhazur R. Sarker^1^; Elizabeth Nicole Teal^1^; Marni B. Jacobs^2^; Louise C. Laurent^3^; Natalie A. Bello^4^; Timothy Wen^5^; Russell S. Miller^6^; Cynthia Gyamfi‐Bannerman^1^

*^1^University of California, San Diego, San Diego, CA; ^2^University of California, San Diego Health, San Diego, CA; ^3^University of California, San Diego Medical Center, La Jolla, CA; ^4^Cedars Sinai Medical Center, Los Angeles, CA; ^5^University of California, San Diego, Irvine, CA; ^6^Columbia University Medical Center, New York, NY*

2:00 PM ‐ 2:15 PM

**Objective**: Though de novo postpartum hypertension (dnPPHTN) accounts for up to two thirds of PPHTN cases, the physiologic and pathophysiologic cardiovascular changes after delivery are poorly understood. We sought to define longitudinal PP blood pressure (BP) patterns in patients at risk for PPHTN.

**Study Design**: We analyzed data from a negative randomized trial (PMID 38641089) of 82 normotensive patients at high risk for dnPPHTN randomized to daily furosemide or placebo from PP day 1‐5. BPs were monitored every 4‐8 hours from delivery to discharge, then by Bluetooth‐enabled remote monitoring twice‐daily for 6 weeks. The primary goal of this secondary analysis was discovery of distinct patterns of longitudinal BP trajectories in the placebo group. Secondary goals included exploring differences in early PP BP trends between those who developed dnPPHTN and those remaining normotensive; and timing of peak BPs. Trends were assessed using local polynomial regression fitting. Linear mixed‐effects models were used to examine temporal BP trajectories, including polynomial time effects, linear spline, and random intercepts and slopes. An interaction term for the time trend and dnPPHTN diagnosis was included.

**Results**: We included all 40 placebo participants from the parent trial, contributing 2235 PP BP readings. SBP and DBP rose until days 10 and 12 PP, respectively, before declining. Mean peak SBPs and DBPs were 120.7 ± 13.4mmHg and 81.0 ± 10.3mmHg. There were significant differences in BP trajectories between participants with (n = 3; 167 BP readings) and without (n = 37; 2068 BP measurements) dnPPHTN (**Figure 1**). Those with dnPPHTN had a significantly steeper rise in SBP preceding dnPPHTN diagnosis, which occurred at a median of 5 days [IQR 5,5.5 days]. SBPs rose by 1.6mmHg/day more to its PP day 9 peak in dnPPHTN compared to normotensive participants, while DBP rose by 0.3mmHg/day more and peaked later (PP day 14 vs 12) (**Table 1**).

**Conclusion**: Using innovative technology, we defined distinct trends in PP cardiovascular physiology that distinguish physiologic from pathophysiologic BP changes in patients at risk for dnPPHTN.

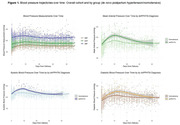

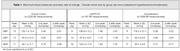

## 60 | Severe Systolic Hypertension and Adverse Maternal Outcomes

Yossi Bart^1^; Hector M. Mendez‐Figueroa^2^; Farah H. Amro^1^; Baha M. Sibai^1^

*^1^McGovern Medical School at UTHealth Houston, Houston, TX; ^2^McGovern Medical School at UTHealth, Houston, TX*

2:15 PM ‐ 2:30 PM

**Objective**: Severe hypertension (sHTN) in pregnancy is associated with maternal morbidity. The specific role of sHTN based on isolated severe systolic blood pressure has not been well studied. We aimed to examine whether sHTN diagnosed by elevated systolic BP (SBP) alone is associated with adverse maternal outcomes.

**Study Design**: We conducted a secondary analysis of the Assessment of Perinatal Excellence (APEX) database. We included all the patients who had hypertension at the time of delivery, defined as SBP ≥ 140 mmHg or diastolic BP ≥ 90 mmHg on two occasions at least 30 minutes apart. We excluded individuals with diastolic BP ≥ 110 mmHg. Our reference group were those with mild HTN, defined as: systolic BP 140‐159 and/or diastolic BP 90‐109. We had 3 comparison groups; group I: SBP 160‐179, group II: SBP 180‐199, and group III: SBP > 200 mmHg. The primary outcome was defined as a composite of maternal outcomes, including hypertensive stroke, pulmonary edema, acute kidney injury, disseminated intravascular coagulation, cardiopulmonary arrest, and death. Poisson regression was applied to address possible confounders.

**Results**: Overall, 38,245 individuals met the inclusion criteria, of which 32,277 (84%) had mild HTN and 5,968 (16%) had severe systolic HTN. Of those, 3,790 (63%) had SBP 160‐179, 1,903 (32%) had SBP 180‐199, and 275 (5%) had SBP > 200 mmHg. Higher SBP was associated with higher rates of advanced maternal age, obesity, preterm birth, and chronic HTN. Following adjustment, higher systolic BP was associated with higher rates of the composite outcome, driven mainly by higher rates of pulmonary edema and acute kidney injury. The relative risk for composite maternal outcome was 2.93 (95% CI 2.24‐3.85) for SBP 160‐179 mmHg, 3.92 (95% CI 2.88‐5.33) for SBP 180‐199, and 6.74 (95% CI 4.01‐11.31) for SBP ≥ 200 mmHg. Further stratification to 10 mmHg intervals of SBP demonstrate the dose‐dependent association with the composite outcome (Figure).

**Conclusion**: Compared to mild HTN, severe HTN diagnosed by SBP alone was associated with maternal morbidity in a dose‐dependent fashion.

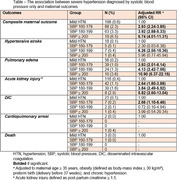

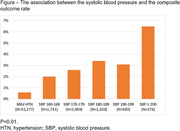

## 61 | Postpartum Diuretics for Hypertensive Disorders of Pregnancy: A Systematic Review and Meta‐Analysis

Emma Trawick Roberts^1^; Elana Jaffe^1^; Johanna Quist‐Nelson^1^; Suneet Chauhan^2^; Baha M. Sibai^3^; Michal Fishel Bartal^4^

*^1^University of North Carolina, Chapel Hill, NC; ^2^Christiana Care, Newark, DE; ^3^McGovern Medical School at UTHealth Houston, Houston, TX; ^4^UTH Houston & Sheba Medical Center Israel, Houston, TX*

2:30 PM ‐ 2:45 PM

**Objective**: To systematically review postpartum use of diuretics to reduce persistent hypertension (HTN) among individuals with hypertensive disorders of pregnancy (HDP).

**Study Design**: A systematic review was conducted in MEDLINE, OVID, Scopus, World Health Organization International Clinical Trials Registry Platform, ClinicalTrials.gov and the Cochrane Central Register of Controlled Trials from inception to 4/2024 (PROSPERO CRD42021288277). Randomized controlled trials (RCTs) investigating postpartum use of diuretics in individuals with HDP were included. Clinical characteristics and outcome measures were extracted. The primary outcome was persistent HTN postpartum. Secondary postpartum outcomes were use of additional antihypertensive drugs, length of stay, readmission/emergency visits, and breastfeeding. Meta‐analysis was performed using a random effects model to estimate treatment effects in terms of risk ratios (RR) or mean difference with 95% confidence interval (CI).

**Results**: Of 571 studies reviewed, eight RCTs with 1368 individuals (767 intervention, 771 control) met inclusion criteria. Interventions included furosemide (3 RCTs), torsemide (1 RCT), combined furosemide/antihypertensive drug (3 RCTs), and combined thiazide/angiotensin‐converting enzyme inhibitor (1 RCT). There was a significant reduction in persistent HTN between intervention and control groups (4 RCTs, N = 955, RR 0.53, 95%CI 0.35‐0.80, I^2^ = 70%, Figure 1). The number needed to treat (NNT) to reduce persistent HTN was 4. There was no difference in the need for additional antihypertensives during admission (7 RCTs, N = 1209, RR 0.79, 95%CI 0.61‐1.02, I^2^ = 7%) or at the time of discharge (5 RCTs, N = 905, RR 1.00 95%CI 0.82‐1.21, I^2^ = 53%). There was no difference in length of stay, readmission/emergency visits, or breastfeeding between groups.

**Conclusion**: Diuretics were associated with a reduction in persistent HTN postpartum in individuals with HDP (NNT = 4). Heterogeneity in study interventions and outcomes suggests that the optimal postpartum dosing and timing of diuretics in HDP warrants further study.

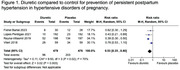

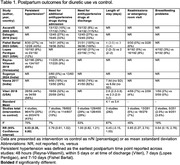

## 62 | Association of Preeclampsia with Long‐Term Risk of Neurodegenerative Disorders

Maria Bazan^1^; Ai‐ris Y. Collier^2^; Tina Yi Jin Hsieh^2^; James Cheng‐Chung Wei^3^

*^1^Beth Israel Deaconess Medical Center, Brighton, MA; ^2^Beth Israel Deaconess Medical Center, Boston, MA; ^3^Chung‐Shan Medical School, Taichung, Taichung*

2:45 PM ‐ 3:00 PM

**Objective**: Preeclampsia (PE) is a common complication of pregnancy linked to increased lifetime risk of cardiovascular disease. The link between PE and development of adverse neurologic conditions is poorly understood. Our objective was to describe the rates of neurodegenerative disorders occurring 3 or more years following complications from PE compared to those without PE.

**Study Design**: We utilized the TriNetX database including diagnoses codes from 66 healthcare organizations to identify women 18+ years old with last birth between 1/2000‐12/2023. PE was defined using ICD‐10 codes occurring before delivery up until December 2020. The comparator group included pregnant women in the same period without PE diagnosis. After 1:1 propensity score matching (PSM) on age at index pregnancy, demographics, health utilization, comorbidities, depression, reproductive factors, and BMI, hazard ratios (HR) and 95% confidence intervals (CI) for cognitive disorders were estimated between the PE and comparator group using a Cox proportional hazard regression model. Cognitive outcomes were identified by ICD‐10 codes and censored until one of the following conditions was met: 1) development of an outcome, 2) July 26, 2024, or 3) loss to follow‐up in the system.

**Results**: The cohort included 163,296 pregnant women with PE and 1,647,539 without PE. In those with prior PE, 8,012 (4.9%) developed neurodegenerative outcomes, compared to 46,337(2.8%) in the control group. After PSM, the PE exposure group demonstrated an increased risk of symptomatic cognitive impairment outcomes (HR 1.4, CI 1.351‐1.457), Parkinson's disease (HR 1.389, CI 1.035‐1.864), Parkinsonism (HR 1.242, CI 1.059‐1.455), and combined Parkinson's disease and Parkinsonism (HR 1.285, CI 1.115‐1.481) compared to those without PE (Table 1).

**Conclusion**: Our findings show a higher risk of cognitive impairment, Parkinson's disease, and Parkinsonism in those with a history of PE. Obstetrical health is a window to lifelong neurocognitive health. This study supports enhanced surveillance and monitoring of neurodegenerative diseases.

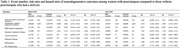

## 63 | Comprehensive Blood Pressure Management to Improve Early Postpartum Blood Pressure Parameters: a Randomized Controlled Trial

Alexandra JD Phelps^1^; Loveis Jackson^1^; Lilly He^1^; Ashanti E. Griggs‐Cooks^1^; Neha Dudipala^2^; Etoi Garrison^1^; Kathryn J. Lindley^1^; Soha S. Patel^1^; Julia C. Phillippi^3^; Megan Webb^1^; Sarah S. Osmundson^1^

*^1^Vanderbilt University Medical Center, Nashville, TN; ^2^Ohio State University College of Medicine, Columbus, OH; ^3^Vanderbilt University School of Nursing, Nashville, TN*

3:00 PM ‐ 3:15 PM

**Objective**: To determine whether comprehensive vs clinician‐directed blood pressure (BP) management and tight vs standard BP control improves BP parameters at 7‐10 days postpartum.

**Study Design**: Postpartum patients with cHTN, gHTN, or preeclampsia were randomized to two interventions (four arms): 1) comprehensive BP management with remote monitoring, daily BP reporting, and algorithm‐based treatment by an MFM‐supervised nurse practitioner vs clinician‐directed BP monitoring and 2) tight (goal BP < 140/90) vs standard (goal BP < 150/100) control. All participants received a BP cuff, directions how to monitor BPs twice daily, and instructions to attend an in‐office BP check 7‐10 days postpartum. The primary outcome was in‐office BP check. Secondary outcomes included reporting any BPs either remote or in person, mean BPs, and the frequency of BPs > = 140/90 or > = 160/110 within 7‐10 days postpartum.

**Results**: Between 5/9/2023 and 7/26/2024, 644 patients were eligible, 327 (51%) enrolled and were randomized, and 323 participants available for the final analysis after 4 withdrawals. Demographic and clinical characteristics of the population are presented in Table 1. Comprehensive management increased the frequency of attending an in‐office, 7‐10 day BP check compared to clinician management, but the results were not statistically significant (62% vs 51%, aOR 1.53 [0.94‐2.50]). Comprehensive management significantly increased the frequency of reporting any BP either remote or in person (94% vs 85%, aOR 2.43 [1.05‐5.63]).  Findings did not vary by race or insurance status. Other secondary outcomes were not statistically significant and were unchanged after accounting for interaction between the two interventions.

**Conclusion**: Compared to clinician‐directed management, comprehensive BP management did not increase the frequency of attending an in‐office BP check but did increase the frequency of reporting any BP between 7‐10 days. These findings suggest a comprehensive program to intensely monitor BPs in the early postpartum period increases patient engagement with BP management.

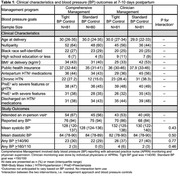

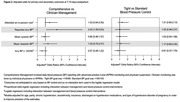

## 64 | Hofbauer Cells May Mediate PE‐Associated Placental Dysfunction via APOE Synthesis

Bani Medegan Fagla; Cielo Dela Rosa; Savita Sundar; Aswathi Jayaram; Erykah Walton; Jason York; Leon M. Tai; Guomao Zhao; Irina A. Buhimschi

*University of Illinois at Chicago, College of Medicine, Chicago, IL*

3:15 PM ‐ 3:30 PM

**Objective**: Preeclampsia (PE) was recently described as a protein misfolding disorder of pregnancy sharing pathophysiological processes with Alzheimer's disease (AD). Among these, amyloid‐beta (Aβ) accumulation, characteristic for AD brain, was also identified in PE placenta. Apolipoprotein E (ApoE), a key lipid regulator synthesized in the brain by astrocytes, neurons, and microglia mediates Aβ accumulation and clearance. To our knowledge, in situ synthesis of ApoE mRNA by human placental cells has not been investigated. Herein, we aimed to identify ApoE producing cells in the human and mouse placenta.

**Study Design**: Immunohistochemistry (IHC) was performed on human placentas from pregnancies with (n = 10, GA: 30.6±3.3 wks) and without (n = 9, GA: 33.6±3.3 wks) PE. Placentas of humanized *ApoE* transgenic mice were also studied (n = 16 mice). Dual RNA in situ hybridization (ISH)+IHC was conducted to localize ApoE RNA relative to macrophage markers CD163 (human) and F4/80 (mouse). In transgenic mice, we also quantified F4/80 signal intensity after Reduced Uteroplacental Perfusion Pressure (RUPP), a validated surgical procedure that induces PE‐like symptomatology. Control mice underwent sham procedures (n = 5‐11/group).

**Results**: 1) In human placenta, IHC identified ApoE in fetal blood vessels (fbv), basement membrane (bm), villous stroma (vs) and Hofbauer cells (hbc) which stained conspicuously  (Figs. A&B); 2) In mice, ApoE was diffusely present throughout the labyrinth (lab) and decidua (de) (Fig. C); 3) In human placenta, ApoE mRNA was selectively present in Hofbauer cells identified by co‐expression of CD163 (Fig. D&E); 4) Similarly, in mice, ApoE mRNA co‐localized with the macrophage marker F4/80 (Fig. F); 5) F4/80 expression decreased in response to RUPP in mouse placenta (p = 0.001 vs. sham).

**Conclusion**: Placental HBCs, which are known to share common embryologic origin with brain microglia, synthesize ApoE and may contribute to clearance or accumulation of misfolded proteins in the placenta and thus to PE pathogenesis.

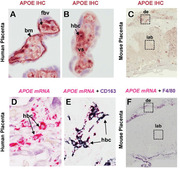

## 65 | Impact on Severe Maternal Morbidity following Statewide Improvements in Hypertensive Disorders of Pregnancy Care Processes

Patrick Schneider^1^; Allison Stevens^2^; Alyssa Antonini^2^; Michelle Menegay^3^; Allison Lorenz^3^; Stephen Afflito^3^; Rashelle Ghanem^4^; Ryan Everett^5^; Justin R. Lappen^6^

*^1^The Ohio State University, Wexner Medical Center, Columbus, OH; ^2^Government Resources Center for the Ohio Colleges of Medicine, Columbus, OH; ^3^Ohio Colleges of Medicine, Government Resource Center, Columbus, OH; ^4^Ohio Department of Children and Youth, Columbus, OH; ^5^Ohio Hospital Association, Columbus, OH; ^6^Cleveland Clinic Foundation, Cleveland, OH*

3:30 PM ‐ 3:45 PM

**Objective**: Hypertensive disorders of pregnancy (HDP) are a major cause of severe maternal morbidity (SMM). While statewide quality improvement (QI) projects have significantly improved HDP care processes, their impact on SMM remain unclear.

**Study Design**: As part of a statewide QI project initiated during the COVID‐19 pandemic, 30 maternity hospitals implemented an HDP care bundle which included timely treatment of severe hypertension (HTN) and subsequent care management. Monthly patient data was collected on all new cases of sustained severe HTN. Hospital SMM rates defined by CDC criteria were obtained from the state hospital association. Data were analyzed for periods before (10/2018‐9/2020) and after (10/2020‐9/2023) implementation. R (4.3.3) was used to conduct a controlled interrupted time series analysis. The primary outcomes were the difference in HDP SMM trends overall, by race, and Medicaid status at participating hospitals before/after bundle implementation. Secondary outcomes included the difference in HDP SMM trends overall, by race, and Medicaid‐status at participating vs. non‐participating hospitals and change in timely treatment of severe HTN among participating hospitals before/after bundle implementation.

**Results**: Timely treatment increased after bundle implementation (baseline 56.9% to 77.1%; p< 0.001).  Though the increase in rates appeared attenuated, no statistically significant change in the overall, Black, and Medicaid HDP SMM rates before/after implementation were found (p = 0.43; 0.67; 0.82; Table 1). Similarly, no statistically significant change in the overall, Black, and Medicaid HDP SMM rates among participating vs. non‐participating hospitals before/after implementation were found (p = 0.52; 0.30; 0.46; Figure 1).

**Conclusion**: In this statewide HDP QI project, no significant changes in HDP SMM rates were detected despite improvements in HDP care processes. While this demonstrates that improvements in HDP care processes may not be the sole driver of HDP SMM reductions, it supports further exploration of non‐clinical and contextual factors impacting SMM, such as the COVID‐19 pandemic.

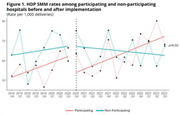

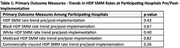

## 66 | Adverse Outcomes During Delivery Hospitalizations Among Patients with an Intellectual or Developmental Disability Diagnosis

Manasa G. Rao^1^; Timothy Wen^2^; Mary E. D'Alton^1^; Alexander M. Friedman^3^; Noelia Zork^3^

*^1^Columbia University Medical Center, New York, NY; ^2^University of California, San Diego, Irvine, CA; ^3^Columbia University Irving Medical Center, New York, NY*

3:45 PM ‐ 4:00 PM

**Objective**: People with intellectual and developmental disabilities (IDD) have higher risk for obesity, diabetes, asthma, and cardiovascular disease. There is limited contemporary data on pregnancy outcomes in the setting of IDD. The objective of this study was to evaluate whether IDD is associated with adverse perinatal outcomes in a large national sample.

**Study Design**: Delivery hospitalizations to patients aged 15‐54 were analyzed using the 2000‐2021 Nationwide Inpatient Sample. Primary exposure of interest was IDD, defined using ICD9/ICD10 codes. Temporal trends in proportion of deliveries with IDD were analyzed with joint point regression to determine average annual percent change (AAPC) with 95% confidence intervals. Adjusted logistic regression models were performed for outcomes accounting for hospital, demographic, and clinical factors with IDD as the exposure of interest.

**Results**: Of 83.6 million births, 23,347 (0.03%) had an associated IDD diagnosis. Deliveries to patients with IDD increased from 1 per 10,000 in 2000 to 6 per 10,000 in 2021 (AAPC 7.7%, 95% CI: 1.0%‐8.8%, p< 0.01). IDD deliveries were more likely to have a diagnosis of obesity (p< 0.01), asthma (p< 0.01), chronic hypertension (p< 0.01), pregestational and gestational diabetes (p< 0.01), or a mental health condition (p< 0.01). In adjusted analyses, IDD deliveries were more likely to have preterm birth at < 37 (aOR 1.5, 1.3‐1.7), < 34 (aOR 1.7, 1.4‐2.0), < 32 weeks (aOR 1.7, 1.4‐2.1), cesarean delivery (aOR 1.6, 1.5‐1.8), operative vaginal delivery (aOR 1.6, 1.4‐1.8), non‐transfusion severe maternal morbidity (aOR 1.7, 1.4‐2.1), transfusion (aOR 1.34, 1.09‐1.65), hypertensive disorders of pregnancy (aOR 1.2, 1.1‐1.3), postpartum hemorrhage (aOR 1.2, 1.1‐1.4), infection (aOR 1.2, 1.0‐1.4), and stillbirth (aOR 2.3, 1.9‐2.8). Likelihood of placental abruption and infection were not significantly different between groups.

**Conclusion**: IDD among delivery hospitalizations is increasing and is associated with adverse perinatal outcomes. Additional resources and surveillance may be warranted for patients with IDD to optimize outcomes.